# Combining Cold Atmospheric Plasma and Environmental Nanoparticle Removal Device Reduces Neurodegenerative Markers

**DOI:** 10.3390/ijms252312986

**Published:** 2024-12-03

**Authors:** Nerea Menéndez-Coto, Claudia Garcia-Gonzalez, Francisco Javier Baena-Huerta, Rubén Zapata-Pérez, Rubén Rabadán-Ros, Estrella Núñez-Delicado, Lucía González-Llorente, Enrique Caso-Peláez, Ana Coto-Montes

**Affiliations:** 1 Department of Morphology and Cell Biology, University of Oviedo, 33006 Oviedo, Spain; 2 Research Group Oxidative Stress Knowledge and Advanced Research (OSKAR), Instituto de Investigación Sanitaria del Principado de Asturias (ISPA), 33011 Oviedo, Spain; 3 Instituto de Neurociencias del Principado de Asturias (INEUROPA), 33006 Oviedo, Spain; 4 Instituto de Investigación Sanitaria del Principado de Asturias (ISPA), 33011 Oviedo, Spain; 5 UCAM HiTech Sport & Health Innovation Hub, Universidad Católica de Murcia, Guadalupe de Maciascoque, 30107 Murcia, Spain; 6 Departamento de Ciencias de la Salud, Universidad Católica San Antonio de Murcia (UCAM), Avenida de los Jerónimos s/n, Guadalupe de Maciascoque, 30107 Murcia, Spain; 7 System and Precision Medicine, Hospital Covadonga, 33204 Gijón, Spain

**Keywords:** brain, ageing, neurodegeneration, cold atmospheric plasma, nanoparticles, aggresomes, autophagy

## Abstract

Ageing leads to a gradual deterioration of the organs, with the brain being particularly susceptible, often leading to neurodegeneration. This process includes well-known changes such as tau hyperphosphorylation and beta-amyloid deposition, which are commonly associated with neurodegenerative diseases but are also present in ageing. These structures are triggered by earlier cellular changes such as energy depletion and impaired protein synthesis, both of which are essential for cell function. These changes may in part be induced by environmental pollution, which has been shown to accelerate these processes. Cold Atmospheric Plasma (CAP) or atmospheric pressure gas discharge plasmas have shown promise in activating the immune system and improving cellular function in vitro, although their effects at the organ level remain poorly understood. Our aim in this work is to investigate the effect of a device that combines CAP treatment with the effective removal of environmental nanoparticles, typical products of pollution, on the activity of aged mouse brains. The results showed an increase in energy capacity, a reduction in reticulum stress and an activation of cellular autophagic clearance, minimising aggresomes in the brain. This leads to a reduction in key markers of neurodegeneration such as tau hyperphosphorylation and beta-amyloid deposition, demonstrating the efficacy of the tested product at the brain level.

## 1. Introduction

Recent decades have seen an unprecedented increase in global life expectancy, a phenomenon that contrasts sharply with the reality experienced by humankind throughout its evolutionary history. This increase has been driven mainly by advances in medicine, biotechnology and improved living conditions. However, while this phenomenon is of great benefit to society, it also poses a major challenge: longer lifespans do not always mean longer healthy lifespans. Ageing is often associated with chronic diseases and dependencies, especially those associated with age, which reduce the quality of life of older people. Systems must be developed to delay the onset of these dependencies as long as possible. Within this scenario, neurodegenerative diseases associated with ageing, such as Alzheimer’s, Parkinson’s and other forms of dementia, have become a central focus of biomedical research because of their direct relationship with the cognitive and functional decline that occur with age. The structural and functional complexity of the brain makes it one of the most sensitive organs to ageing. As longevity increases, so does the risk of abnormal protein accumulation, oxidative stress and neuroinflammation, processes that contribute to neuronal degeneration and subsequent cognitive and physical decline [1].

Oxidative stress damages macromolecules, propagates injury by promoting inflammation and ultimately leads to neuronal dysfunction in nervous tissue [2,3]. To counteract this, cells (especially post-mitotic cells such as neurons) have quality control mechanisms. Thus, when unfolded or misfolded proteins accumulate in the endoplasmic reticulum (ER), the proteasome attempts to restore proper reticulum function by removing these abnormal proteins from circulation, but if ER stress persists, a multidirectional response known as the unfolded protein response (UPR) is triggered, which reduces protein synthesis, increases chaperone production and enhances the activity of the ubiquitin/proteasome system [4]. If this is not sufficient to deal with protein accumulation, autophagy (in particular chaperone-mediated autophagy and macroautophagy) will intervene to remove these abnormal proteins [5]. During ageing, these response mechanisms often deteriorate, reducing their efficiency and creating a favourable environment for the development of abnormal molecules. This favours the formation of neurodegenerative structures and aggregates of waste products bound together by cytoskeletal elements, known as aggresomes [6,7].

Exposure to air pollution, particularly airborne nanoparticles, poses a major threat to human health, as these ultrafine particles, derived from fossil fuel combustion and other industrial sources, can penetrate deep into the lungs and reach the circulatory system. These particles have been shown to promote oxidative stress, systemic inflammation and damage at the cellular level, factors associated with an increased risk of respiratory, cardiovascular and neurodegenerative diseases [8]. Older people are particularly vulnerable to these nanoparticles due to the accumulation of cellular damage over time, reduced lung capacity and the higher prevalence of comorbidities such as hypertension or diabetes, which increase the incidence of chronic respiratory diseases and premature mortality in areas with high levels of pollution [9]. In addition, recent studies suggest an association between prolonged exposure to nanoparticles and cognitive impairment, which poses an additional risk for the elderly [10].

Active systems capable of acting directly on the individual are revolutionising preventive approaches to individual health. Thus, atmospheric gas discharge plasmas, in particular those operating in an energy imbalance and at low gas temperatures, known as Cold Atmospheric Plasma (CAP), have recently gained much attention for their potential applications in various technologies. Classically used for the treatment of chronic wounds [11,12,13], CAP has a wide range of as yet unexplored applications [14,15,16], placing it at the forefront of preventive medicine. Plasma is an ionised gas resulting from the decomposition of polyatomic gas molecules or the removal of electrons from the different layers of a monoatomic gas. The ionisation of the gas is not the only requirement for it to be considered a plasma: quasineutrality, Debye shielding and plasma frequency are the properties it must have [17,18,19]. Within the plasma category, only plasma in which the particles are not in thermal equilibrium can be called ‘cold plasma’ [20,21]. Although the first decontaminant applications of low-pressure cold plasma date back to the late 1960s, it was not until the mid-1990s that its potential uses in biomedicine were explored through the induction of reactive oxygen and nitrogen species [11,16,22,23], which appeared to mimic early immune defence pathways. Subsequently, their beneficial effect on osseointegration has been demonstrated in dental medicine [24,25,26,27] and, more recently, through the induction of stem cell proliferation [28,29,30,31] without possible cytotoxic or other negative effects [32]. However, most of these studies have been performed using cell cultures [28,33,34,35] and scarcely in animal models [36,37,38], which requires a deeper understanding of their physiological effects under normal or lifelong conditions.

Recently, our group has shown that the removal of environmental nanoparticles in the 0.3–10 µm aerodynamic size range (PM 0.3–10) using BioW devices induces a significant enhancement in protein synthesis and ATP production in peripheral blood mononuclear cells (PBMCs), demonstrating that this system is able to reverse nanoparticle-induced toxicity and promote cell recovery [39]. Recent advances in the combination of nanoparticle removal and CAP have shown that the removal of nanoparticles from the medium allows for a barrier-free laminar flow of anions that could therefore act directly on individuals by penetrating the skin and mucous membranes. On this basis, our intention in this article is to study the effect that this combined equipment can have on experimental animals at the cerebral level. The results obtained not only allowed us to ensure an action of this system on the brain of these individuals but also indicated that the effects studied showed an improvement in the levels of oxidative stress together with a significant reduction in the expression of markers of neurodegeneration associated with ageing, which opens the door to a possible beneficial function at the brain level in aged individuals.

## 2. Results

No weight changes were observed between control and CAP-NR animals before or after exposure (Supplementary Material Table S1), and no behavioural and/or physical abnormalities were observed in the animals.

### 2.1. Oxidative Stress Damage

**Figure 1:**
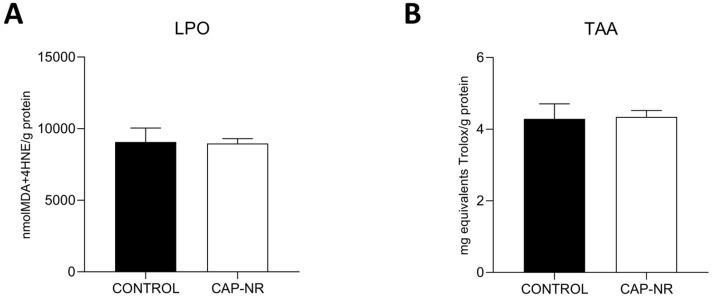
Oxidative stress status in the brain of control and CAP-NR-treated mice. (**A**) Lipid peroxidation (LPO) (expressed as nmol MDA + 4-HNE/g protein); (**B**) total antioxidant activity (TAA) (expressed as mg equivalents Trolox/mL). Data are represented as the mean and standard error of the mean (SEM).

Oxidative damage to proteins was evaluated by measuring the lipid oxidative damage (LPO) and total antioxidant activity (TAA) in mouse brain lysates. No significant differences were found between control animals and CAP-NR-treated animals (Figure 1A,B).

### 2.2. Brain Cytokine Levels

**Figure 2:**
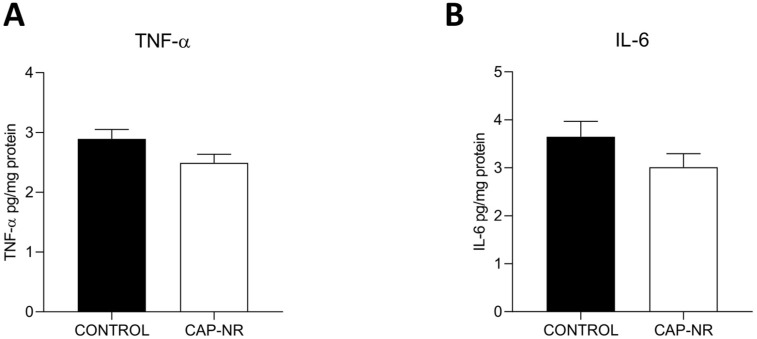
Inflammatory markers in the brain of control and CAP-NR-treated mice. The expression of cytokines is measured as pg IL/mg total protein: (**A**) TNF-α, (**B**) IL-6. Data are represented as mean and standard error of the mean (SEM).

We assessed the levels of TNF-α and IL-6 to determine the impact of CAP-NR treatment on the inflammatory status within the brain. Enzyme-linked immunosorbent assay (ELISA) analysis of mouse brain lysates revealed no significant differences in the levels of these two cytokines (Figure 2A,B).

### 2.3. ATP Levels

**Figure 3:**
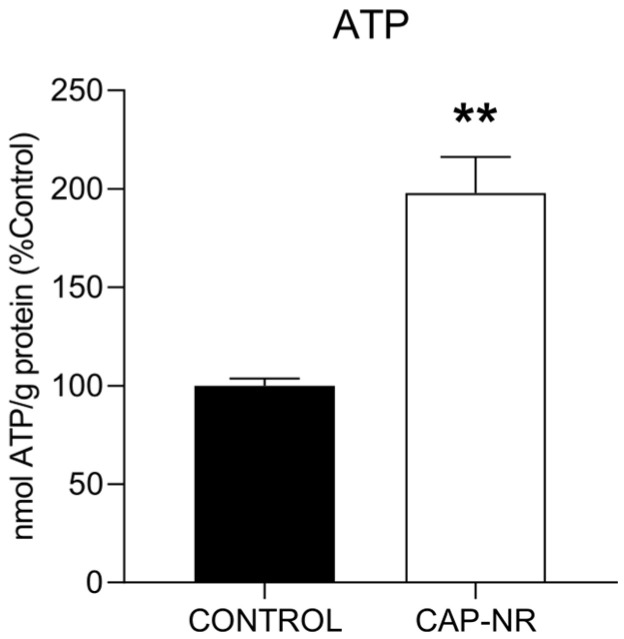
ATP levels in the brain of control and CAP-NR-treated mice. ATP levels are expressed as nmol ATP/g total protein. ** *p* ≤ 0.01. Data are represented as mean and standard error of the mean (SEM).

The levels of ATP serve as a critical indicator of the overall health status of individual cells, with a reduction in ATP being the most prevalent characteristic observed in dying cells. Therefore, we assessed the ATP levels in brain lysates, and we found that CAP-NR-treated mice showed a significant increase in ATP levels compared to age-matched controls (*p* < 0.01) (Figure 3). The regulation of ATP has been reported to mitigate pathological features in murine models of Parkinson’s disease [40], suggesting that sustaining ATP concentrations could play a crucial role in protecting susceptible neuronal cells in the context of neurodegenerative disorders.

### 2.4. Exposure to CAP-NR Decreases ER Stress and Proteasome Activity in the Brain

The pathology associated with numerous neurodegenerative diseases is characterised by the accumulation of misfolded proteins within the brain. Consequently, the mechanisms involved in maintaining proteostasis are crucial for counteracting this detrimental process. The imbalance of protein homeostasis caused by endoplasmic reticulum (ER) stress and the triggering of the unfolded protein response (UPR) has been identified as a critical and widespread pathogenic mechanism in the context of neurodegenerative diseases [41].

**Figure 4:**
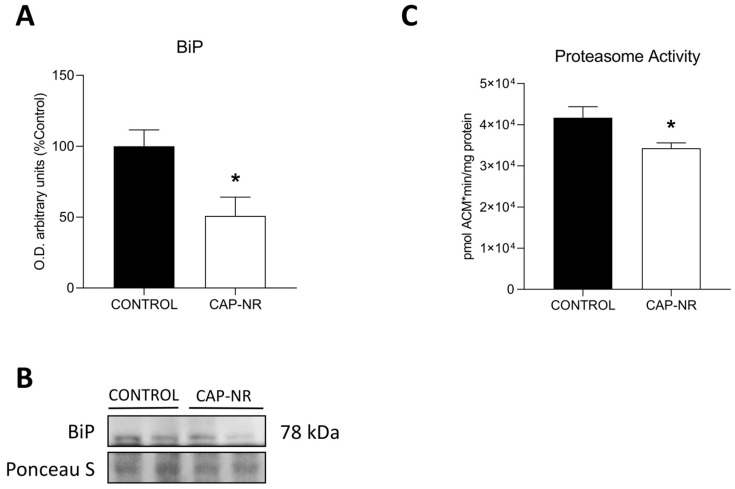
Endoplasmic reticulum (ER) stress and proteasomal activity in the brain of control and CAP-NR-treated mice. Representative immunoblots and quantification of (**A**) BiP; (**B**) quantification; (**C**) proteasome activity ratio (expressed as pmol AMCx min/mg of protein). * *p* ≤ 0.05. Data are represented as mean and standard error of the mean (SEM).

BiP/GRP78, a chaperone that plays a crucial role in maintaining ER quality control, exhibited a marked reduction in expression levels in CAT-NR-treated animals (*p* < 0.05) (Figure 4A,B). The reduction in ER stress in CAP-NR-treated mice indicates a lesser necessity for proteostasis observed in these treated animals.

Multienzyme complexes known as proteasomes are vital for the maintenance of protein homeostasis (proteostasis) and the execution of key cellular functions, primarily through the breakdown of misfolded, excess and damaged proteins. Increased proteasome activity has been associated with the accumulation of damaged and misfolded proteins during the ageing process. In line with the reduction in ER stress, proteasome activity significantly decreased (*p* < 0.05) (Figure 4C). The proteasome serves as the first defence mechanism against accumulating proteotoxic stresses, and its activity was reduced in CAP-NR-treated animals.

### 2.5. UPR Pathway in CAP-NR-Treated Mouse Brains

**Figure 5:**
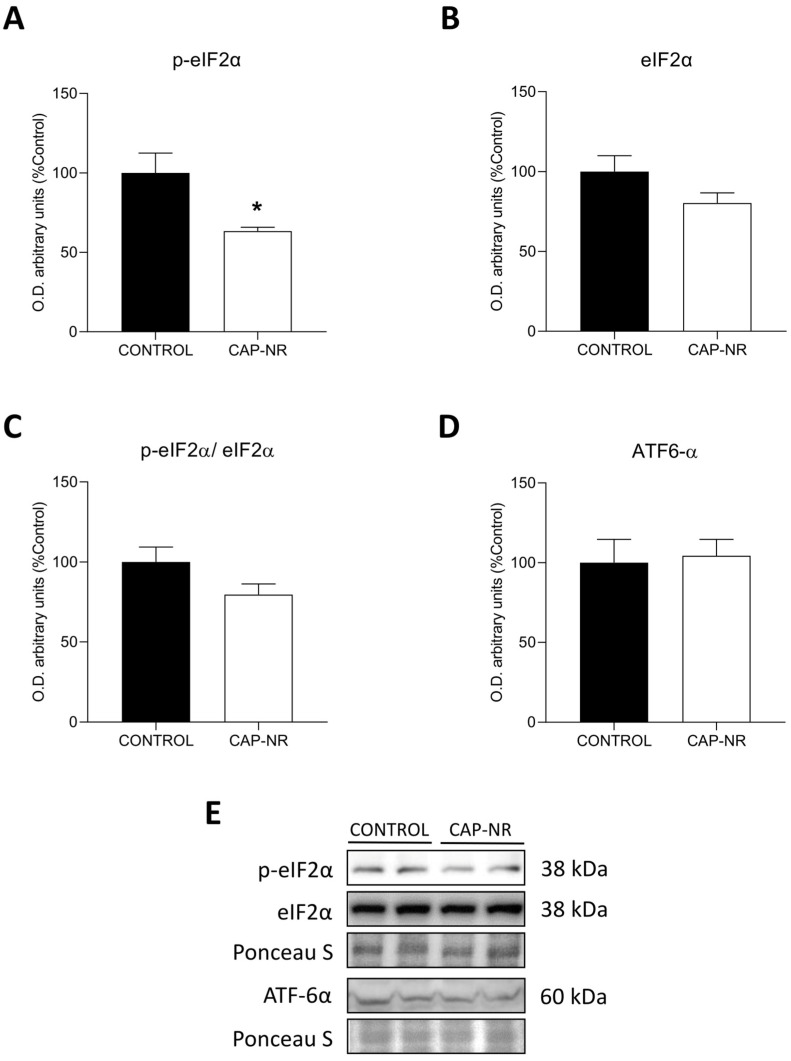
UPR pathway markers in the brain of control and CAP-NR-treated mice. Representative immunoblots and quantification of (**A**) p-eIF2α, (**B**) eIF2α, (**C**) p-eIF2/eIF2 and (**D**) ATF-6α; (**E**) quantification. * *p* ≤ 0.05. Data are represented as mean and standard error of the mean (SEM).

The UPR pathway was evaluated by measuring the PERK pathway represented by the phosphorylation of eIF2α. p-eIF2α (phosphorylated-eukaryotic initiation factor 2α) was found to be significantly decreased in CAP-NR-treated animals compared to controls (*p* < 0.05) (Figure 5A), with no differences found for the total form eIF2α (Figure 5B) or the p-eIF2/eIF2 ratio (Figure 5C), indicating decreased activity of the PERK pathway. In addition, the cleaved and active forms of activating transcription factor 6 (ATF6α) showed no significant differences between groups (Figure 5D).

### 2.6. Autophagy Activation

As a fundamental intracellular process for the breakdown of aggregated proteins and dysfunctional organelles, autophagy has been shown to contribute to the pathological changes observed in numerous neurodegenerative disorders. When autophagy is suppressed, the removal of these harmful substrates is hindered. Conversely, the activation of autophagy can facilitate the improved clearance of these toxic proteins. To determine whether protein quality control mechanisms were implicated, the autophagy markers lysosome-associated membrane protein type 2a (LAMP-2A), Beclin-1, p62 and LC3 I and II were evaluated.

**Figure 6:**
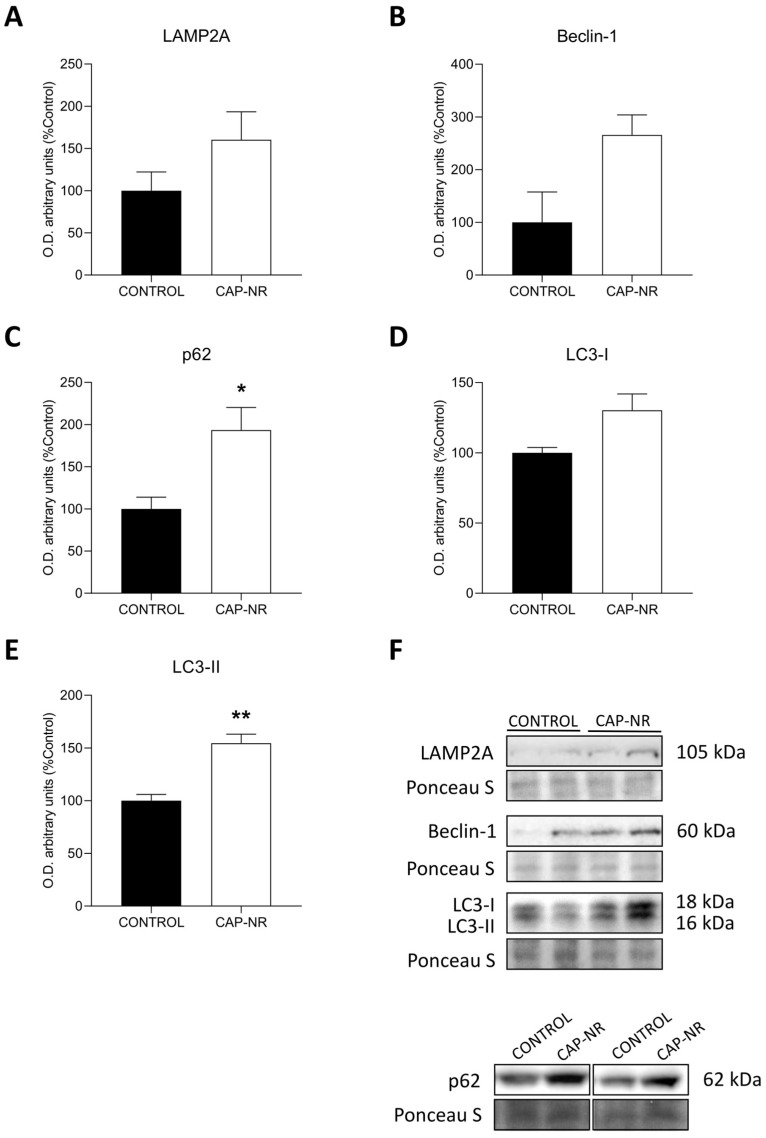
Autophagy markers in the brain of control and CAP-NR-treated mice. Representative immunoblots and quantification of (**A**) LAMP2A, (**B**) Beclin-1, (**C**) p62, (**D**) LC3-I and (**E**) LC3-II; (**F**) quantification. * *p* ≤ 0.05, ** *p* ≤ 0.01. Data are represented as mean and standard error of the mean (SEM).

Immunoblots comparing protein expression levels of LAMP2A (Figure 6A) and Beclin-1 (Figure 6B) showed non-significant differences between brain lysates from CAP-NR-treated animals and those from control animals. Nonetheless, the expression levels of p62 were significantly increased in CAP-NR-treated animals (*p* < 0.05) (Figure 6C), as well as those of LC3-II (*p* < 0.01) (Figure 6E), while LC3-I levels presented non-significant differences (Figure 6D). This increased expression of macroautophagy markers indicates a potential neuroprotective effect reducing the levels of damaged and aggregated proteins as an adaptive cellular process aimed at preserving protein stability.

### 2.7. CAP-NR Treatment Decreases Neurodegeneration Markers

**Figure 7:**
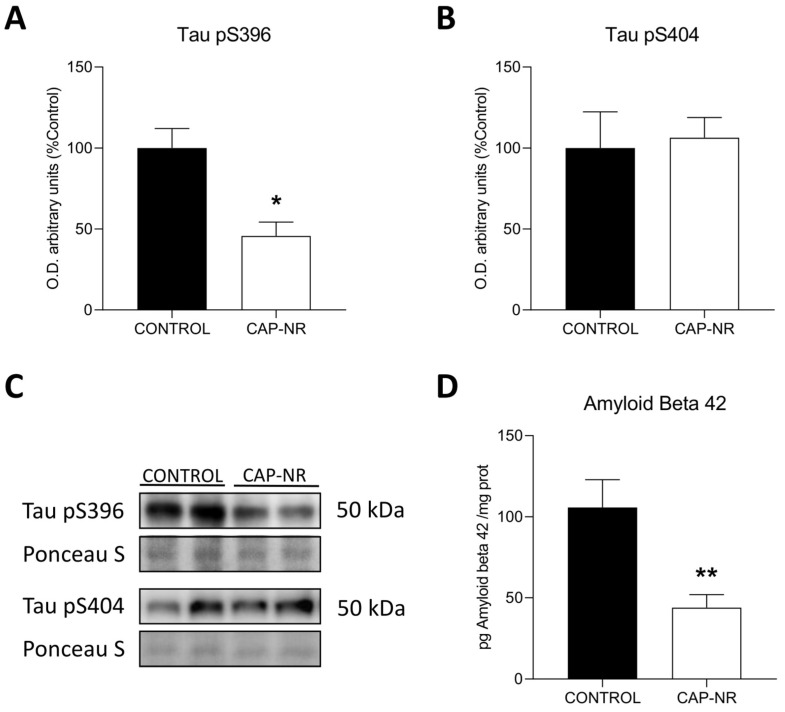
Expression of neurodegenerative markers in the brain of control and CAP-NR-treated mice. Representative immunoblots and quantification of (**A**) Tau pS396 and (**B**) Tau pS404; (**C**) quantification; (**D**) expression of Aβ42 measured as pg/mg total protein. * *p* ≤ 0.05, ** *p* ≤ 0.01. Data are represented as mean and standard error of the mean (SEM).

To explore the effects of CAP-NR on the mouse brain, we initially assessed the expression of early response neurodegeneration markers associated with Alzheimer’s disease [42] in control and CAP-NR-treated mice. We initially evaluated tau epitopes that are commonly phosphorylated in neurodegenerative diseases, Tau pS396 and Tau pS404. We found a lower expression of Tau pS396 (*p* < 0.05) (Figure 7A) in CAP-NR-treated mice brains and no significant changes in Tau pS404 (Figure 7B). Additionally, amyloid beta 42 (Aβ42), a well-known biomarker for Alzheimer’s disease that is linked to age-related neurodegeneration, showed a significantly decreased expression as measured by ELISA in brain lysates from CAP-NR-treated mice compared to that in the age-matched control group (*p* < 0.01) (Figure 7D). These results indicate that exposure to CAP-NR decreases the expression of neurodegenerative markers Tau pS396 and Aβ42, which play a crucial role in tauopathies and Alzheimer’s disease.

## 3. Discussion

Scientific research on ageing has mostly focused on delaying the onset of dependency, preventing age-related diseases and developing effective preventive strategies. In this context, therapeutic and preventive approaches have been explored, such as the early identification of biomarkers and interventions in modifiable lifestyle factors that could delay neurological decline [43]. The main goal of this research is not only to prolong life but also to improve the quality of life for older people, delaying the onset of dependency and promoting healthy ageing. In this sense, current studies advocate a preventive and personalised approach capable of identifying individual risk factors in order to intervene specifically in each patient, a key paradigm in personalised medicine [44].

Although the beneficial effects of CAP are increasingly documented, the question of whether this laminar flow of ions can induce oxidative stress in cells exposed to it has been raised before [45,46]. Any increase in oxidative stress is associated with subsequent oxidative damage if the antioxidant status remains unchanged. This is particularly important in the case of the brain, which is known to have a poor antioxidant defence system [47]. Therefore, the first aim of our study was to determine the existing oxidative damage status. The results showed no significant differences in oxidative lipid damage; furthermore, the levels of antioxidant defence remained stable and were not increased by the equipment used, so we can conclude that the levels of oxidative stress remained within the normal range. This is particularly relevant, as lipid dysregulation in the brain has recently been linked to neurodegeneration and the progression of neurological pathologies [48].

While increased oxidative stress, in general, is associated with an increased immune response [49], in the case of the brain, an excessive immune response could lead to brain inflammation, which is not without risk [50], especially if its effects are limited to an ageing population [51], so this increased immune response would also be undesirable as a response to bioequipment exposure. However, there is conflicting literature on the effects of CAP on inflammation, with both pro-inflammatory [45] and anti-inflammatory [52] effects documented, making this study necessary. In our case, and in line with the levels of oxidative damage observed, there were no significant differences or worrying trends in the levels of the main cytokines responsible for triggering the immune response—IL-6 and TNF-α—at the brain level between the two study groups, so inflammation remained stable, and consequently, inflammation could not be adduced as a cause or effect of the other results observed in this study.

Cellular function is fundamentally based on adequate energy production and maintenance and proper protein synthesis. Both cellular pillars are significantly affected by ageing [53,54], with the brain being one of the most affected organs, as decreasing energy capacity is at the basis of neurodegeneration processes [55]. However, no pathological cases associated with energy overproduction have been described. The finding of such a high increase in ATP levels as that shown in the CAP-NR group can only be related to improved mitochondrial function and efficient metabolic activity. This is particularly noteworthy, as there are several articles showing the ability of CAP to provoke ATP depletion in cancer cells [56,57], inducing clear mitochondrial damage, which in no case was manifested in the brain of CAP-NR animals, where the increase was highly significant. The CAP delivery system of the BioW device, which functioned in a constant manner and without contact with the receptor, should be the key differentiating factor.

Protein synthesis, on the other hand, is mainly carried out in the endoplasmic reticulum. It is a fragile process that can be easily disrupted and altered. Factors such as viral or bacterial infections [58,59] and the development of neurodegenerative diseases [20,21] put stress on the ER, a common cellular response in these pathologies. Ageing induces dysregulation of nutrient demand signalling and accumulation of damage in the proteome, ultimately affecting the quality of protein synthesis [53]. Specifically in the brain, this proteomic alteration may underlie the development of several neurodegenerative processes [60], and the enhancement of reticulum stress minimisation mechanisms, such as the UPR, appears to be a suitable therapy to reduce these processes [61]. BiP, an endoplasmic reticulum-resident chaperone involved in the correct folding of proteins, activates the UPR by acting as a marker of rough endoplasmic reticulum stress [62]. Our results show that the BioW device reduced BiP levels, implying a reduction in ER stress, and this was confirmed by the results observed for the proteasome, which is responsible for the standard degradation of abnormal ER proteins and which also showed reduced activity in the CAP-NR group of mice. Recently, the possible role of CAP as an inducer of protein synthesis has been suggested [36]. Our group, on the other hand, has found an improvement in protein synthesis in humans by drastically reducing the inhalation of environmental nanoparticles [39]. Both advances justify the observed results and confirm the ability to restore control of protein synthesis, a rather complex process that has only been demonstrated in a few cases, such as through the use of melatonin, a potent antioxidant with an ability to restore the function of the reticulum that has been widely documented [63].

The activation of the UPR is directly dependent on the level of endoplasmic reticulum stress present, so the observed reduction in the PERK pathway, which is dependent on eiF2-alpha phosphorylation and is the non-adaptive pathway triggered by the most severe endoplasmic reticulum stress, again demonstrates the clear improvement in protein synthesis achieved. This effect at the proteomic level should not be taken lightly, as significant benefits have been demonstrated in the treatment of neurodegenerative diseases by therapies aimed at improving protein synthesis [61].

These observed improvements would be diluted in their beneficial effect if a cellular clearance system was not simultaneously maintained to ensure the removal of damaged particles and toxic aggregates that interfere with cellular transport and efficiency. Of particular interest is the improvement in the autophagic process observed in the brains of CAP-NR mice. The reduction in autophagic capacity is directly involved in the cellular changes associated with ageing [64] and plays a crucial role in maintaining brain homeostasis and preventing the appearance of aggresomes [65], toxic accumulations in the brain that are repeatedly observed in the triggering of neuronal diseases associated with ageing [66]. The other results observed in line with this complete and confirm the effect observed. The increase in p62, a robust marker of protein aggregation [67], which is recognised in autophagy, together with the significant increase in the autophagy marker par excellence, LC3-II, confirm the protection against these neurodegenerative aggregates, which could serve as outlines of neurodegeneration markers.

Several markers of neurodegeneration have been identified. However, tau hyperphosphorylation, initially thought to be an exclusive indicator of Alzheimer’s disease [68] and now known to be present in neuronal damage that may be associated with ageing [69,70], is one of the most important and best studied. Its significant reduction in the brain of CAP-NR mice is therefore undoubtedly a good indicator of the improvement induced in this organ. The beta-amyloid peptide precursor protein, for its part, follows a parallel pathway to tau hyperphosphorylation, having first been associated with Alzheimer’s disease [71] and then extended to the neurodegenerative processes that can accompany ageing [70]. It was also significantly reduced in the brains of CAP-NR mice, underlining the importance of this effect and demonstrating a real preventive or therapeutic capacity of this compound in treating the effects of ageing at the cerebral level. Very few therapies developed so far have shown significant efficacy at the molecular level in the ageing brain [72,73], and their number is drastically reduced if we limit ourselves to external treatments, without drugs or direct contact with the subject, thus reducing possible associated side effects.

The application of CAP in biomedicine is still a very recent technology [15,16], but it has demonstrated extensive beneficial effects in vitro [28,33,35,74,75] and in experimental animals [36,38,76], although studies on its role in humans are very scarce and have so far been practically limited to the fields of dentistry [25,26,27,77], sterilisation [78,79,80] and skin treatment [81]. On the other hand, research on nanoparticle filtration has hardly focused on the study of cellular modifications [82], although its role in the prevention of a large number of pathologies has been widely demonstrated [15]. Therefore, based on the evidence presented for the first time in this article of the effect of the combination of both methods, CAP and NR, on experimental animals, opens the door to a wide range of possible applications in the field of prevention and health, targeting ageing in general and the brain in particular, one of the organs most sensitive to the passage of time and which still requires therapies and technologies aimed at its protection. Let us bear in mind that the combination of both procedures allows, through the elimination of environmental nanoparticles, the laminar flow transmission of anions that directly affect the individual. This new and experimental approach has led to a substantial improvement at the brain level based on a reduction in oxidative stress associated with an improvement in protein synthesis together with an activation of cellular cleaning in an area of greater energetic capacity, all of which are key phases of cellular ageing at the brain level. The resulting improvement is evident in the reduction in aggresomes, tau hyperphosphorylation and beta-amyloid production.

These results represent a breakthrough in the prevention of unwanted ageing defects in the brain and open the door to new and very interesting research

## 4. Materials and Methods

### 4.1. Animals

Fifteen 19-month-old male wild-type (C57BL/6J) mice were purchased from Charles River Laboratories (Charles River Laboratories España, SA, Barcelona, Spain). The mice were maintained with ad libitum access to food and water and were divided into two groups: a control group (CONTROL) consisting of 5 mice and an experimental group to study the combined effect of the broad-spectrum nanoparticle removal (NR) unit and CAP (cold atmospheric plasma), developed by BioW^®^, that was denoted the CAP-NR group (CAP-NR), consisting of 10 mice that were exposed to the BioW device for 7 weeks. The system works constantly for both nanoparticle removal and cold atmospheric plasma. After this period, the animals were sacrificed by exsanguination through the retro-orbital venous sinus under 4% isofluorane anaesthesia, and the brain was removed from each mouse and frozen at −80 °C until use. A total of 5 mice were used in the control group, while the experimental group consisted of 10 mice for all experiments performed.

Animal health was assessed every two days, looking for signs of abnormal behaviour, including weight loss, lethargy and/or physical abnormalities. Animals were weighed before and after 7 weeks of exposure.

The experimental protocol was approved by the Animal Care and Use Committee of the San Antonio Catholic University of Murcia under license A13220911.

### 4.2. Tissue Collection and Homogenisation

The whole brain of each mouse was homogenised using an Ultra-Turrax T25 mixer (IKA, Staufen, Germany) at 4 °C in buffer containing 50 mM sodium phosphate buffer at pH 7.5, 100 mM NaCl, 1 mM Na_3_VO_4_, 1 mM NaF and 1% Triton; 0.9 mL of buffer was used per 100 mg of tissue. The homogenised tissue was centrifuged at 900× *g* for 6 min at 4 °C, and the supernatant containing the proteins was collected and frozen at −80 °C for future use. Protein concentrations in brain homogenates were determined using the Bradford method.

### 4.3. CAP-NR Device

The broad-spectrum nanoparticle removal (NR) unit combined with CAP (cold atmospheric plasma), developed by BioW ^®^, was designed and certified according to EN 60601-1-2:2015 and EN60601-1:2006+AC:2010+A1:2013. The combined system is an electrically powered unit designed to produce small, biologically active, negatively charged ion dilutions of reactive oxygen and nitrogen species (RONS) that are emitted into NP-free ambient air via direct air laminar flow [83,84,85] for absorption into the bloodstream via the lungs/skin [86,87]. Spectroscopic measurements in the ultraviolet–visible (UV-VIS) and ultraviolet (UV) range of the light emitted by the plasma were performed to confirm the existence of RONS in the plasma being used. A fibre-optic spectrometer with a wavelength range between 200 to 800 nm (UV-VIS) (B&W TEK Inc. Models Exemplar-LS, Newark, DE, USA) was used to observe the full spectrum and to detect emissions from neutral atoms and molecules, in addition to those from RONS. In this spectrum, it could be seen that the emission in the visible spectrum (400–800 nm) was very low with respect to the emission in the ultraviolet range (200–400 nm). This emission in the ultraviolet region (200–400 nm) implies a relatively high production of RONS (Figure S1). A scheme of the experimental arrangement can be found in Figure S2. The technical data are as follows:

- Supply voltage: 230 V;
- Frequency: 50 Hz;
- Maximum power: 525 W;
- Protection type (EN 606011-1): class 1;
- IP protection degree: IP 21;
- Functioning mode: continuous;
- Functioning temperature: 10–40 °C;
- Relative humidity: 35–75% without condensation;
- Maximum capacity for ion generation: 200,000/cm^3^

### 4.4. Oxidative Stress

#### 4.4.1. Lipid Oxidative Damage

Lipoperoxidation was determined by measuring the reactive aldehyde malondialdehyde (MDA) and 4-hydroxy-2-(E)-nonenal (4-HNE) levels using the colourimetric method described by Gérard-Monnier et al. [88]. Samples were run in triplicate, loading 50 μL per sample, and were normalised to total protein in the sample.

#### 4.4.2. Total Antioxidant Activity

Total antioxidant activity was determined using the 2,2′-azino-bis (ABTS) cation radical method described by Gonzalez-Calvo et al. [89]. Samples were run in triplicate, loading 50 μL per sample, and were normalised to total protein in the sample.

### 4.5. Inflammation Studies

#### 4.5.1. Tumour Necrosis Factor α (TNF-α)

The concentration of TNF-α in brain lysates was determined using a commercially available TNF alpha Human ELISA Kit (Invitrogen Corp., Carlsbad, CA, USA). All assays were performed according to the manufacturer’s protocols, with each individual measurement utilising a sample volume of 100 μL. Results were normalised to total protein in the sample.

#### 4.5.2. Interleukin 6 (IL-6)

The brain lysate concentration of IL-6 was measured using a commercially available enzyme-linked immunosorbent assay (ELISA) kit (Human IL-6 ELISA Kit; Gen-Probe Diaclone SAS, Besancon, France). All assays were performed according to the manufacturer’s protocols, with each individual measurement utilising a sample volume of 100 μL. Results were normalised to total protein in the sample.

#### 4.5.3. Proteasome Activity

The assessment of proteasome activity in brain lysates was conducted utilising the Fluorometric Proteasome Activity Assay Kit (Abcam, Cambridge, UK), following the manufacturer’s instructions, with duplicate assays performed by loading 1 μL per sample at a 1:100 dilution and normalising to the total protein in the sample.

#### 4.5.4. ATP Measurement

The evaluation of ATP levels in brain lysates was performed using the ATP Bioluminescent Assay Kit (FLAA, Sigma-Aldrich, Burlington, MA, USA). The ATP assay was conducted according to the manufacturer’s guidelines, utilising a SIRIUS luminometer (Berthold, Pforzheim, Germany) to detect light emission resulting from the oxidation of D-luciferin catalysed by firefly luciferase, a reaction dependent on ATP consumption. Samples were run in triplicate, loading 50 μL per sample diluted 1:50, and were normalised to total protein in the sample.

#### 4.5.5. Amyloid Beta 42 Measurement

The concentration of amyloid beta 42 in brain lysates were determined using a commercially available enzyme-linked immunosorbent assay (ELISA) kit (Abeta42 ELISA Kit; Mybiosource Southern California, San Diego, CA, USA). All assays were performed in accordance with the manufacturers’ protocols, using 100 μL per sample at a concentration of 2 mg/mL in triplicate.

#### 4.5.6. Western Blot Immunoassay

Western blot immunoassays were performed by loading 50 μg of protein per sample in triplicate following the protocol previously established by our research group [90], with specific information on primary and secondary antibody dilutions provided in the Supplementary Material (Table S2). Samples were integrated and categorised as the control group and CAP-NR group. Primary antibodies were incubated overnight at 4 °C, while secondary antibodies were incubated for 1 h at room temperature. Band optical densities were quantified using GeneTools 4.3.17.0 software (Syngene, Cambridge, UK). Due to observed variations in the levels of housekeeping proteins (GAPDH, β-actin and α-tubulin), Ponceau S staining (P3504, Merck, Darmstadt, Germany) was used to ensure equal protein loading, serving as a control for total protein.

#### 4.5.7. Statistical Analysis

All data are presented as the mean ± standard error of the mean (SEM). Statistical and graphical analyses were performed using GraphPad Prism 8.0 software (San Diego, CA, USA). The normality of the data was assessed using the Shapiro–Wilk test (*p* < 0.05). For datasets following a normal distribution, a Student’s *t*-test for unpaired samples was used to compare the experimental groups, with statistical significance set at *p* ≤ 0.05. In cases where the data did not meet the normality assumption, the Mann–Whitney U-test was applied to assess differences between groups, also with a significance threshold of *p* ≤ 0.05. Statistical significance levels are indicated as follows: * *p* ≤ 0.05, ** *p* ≤ 0.01, *** *p* ≤ 0.001.

## Data Availability

Data are contained within the article and Supplementary Material.

## Supplementary Materials

The following supporting information can be downloaded at: https://www.mdpi.com/article/10.3390/ijms252312986/s1.
