# Supplementary material for: Combining Cold Atmospheric Plasma and Environmental Nanoparticle Removal Device Reduces Neurodegenerative Markers
Source: Int J Mol Sci. 2024 Dec 3;25(23):12986. doi: 10.3390/ijms252312986 (PMC11641247; doi:10.3390/ijms252312986)
Supplement: Supplementary file 1 [file ijms-25-12986-s001.zip › ijms-3274423-supplementary.pdf]

	Initial weight	Average initial weight	SD initial weight	Final weight	Average final weight	SD final weight	Variation	Average variation	SD variation	t-test
C1	47.2	39.05	4.54	48.7	39.22	5.28	1.5	0.26	0.83	0.949
C2	39.4			39.2			-0.2			
C3	40.2			41.1			0.9			
C4	35			34.2			-0.8			
C5	34.7			34.6			-0.1			
CAP-NR1	40	36.73	2.73	38.2	36.91	2.79	-1.8	0.19	2.17	
CAP-NR2	32.6			33.4			0.8			
CAP-NR3	39			38.5			-0.5			
CAP-NR4	35.2			36.6			1.4			
CAP-NR5	41.6			41.2			-0.4			
CAP-NR6	36.9			36.7			-0.2			
CAP-NR7	37.3			36			-1.3			
CAP-NR8	34.1			39.7			5.6			
CAP-NR9	34			31.3			-2.7			
CAP-NR10	37.6			38.6			1			

Table S1. Total body weight in grams of the animals including initial weight, average initial weight and standard deviation, finial weight, average finial weight and standard deviation, variation from initial to final weight, average of the variation and standard deviation and t-test of the variation.

Primary antibody	Dilution	Reference	Manufacture	Secondary antibody	Dilution	Reference	Manufacture
Tau pS396	1:1000	44752ZG	Invitrogen	Anti-Rabbit (IgG)	1:2000	7074	Cell Signaling Techonology
Tau pS404	1:1000	44758ZG	Invitrogen	Anti-Rabbit (IgG)	1:2000	7074	Cell Signaling Techonology
LAMP2A	1:1000	ab18528	Abcam	Anti-Rabbit (IgG)	1:2000	7074	Cell Signaling Techonology
p62	1:500	H00008878-M01	Abnova	Anti-Mouse (IgG)	1:1000	7076	Cell Signaling Techonology
Beclin-1	1:1000	#3738	Cell Signaling Techonology	Anti-Rabbit (IgG)	1:2000	7074	Cell Signaling Techonology
LC3	1:1000	PD014	MBL	Anti-Rabbit (IgG)	1:2000	7074	Cell Signaling Techonology
eIF2α	1:1000	#5324	Cell Signaling Techonology	Anti-Rabbit (IgG)	1:1000	7074	Cell Signaling Techonology
p-eIF2α	1:1000	#3398	Cell Signaling Techonology	Anti-Rabbit (IgG)	1:1000	7074	Cell Signaling Techonology
ATF-6α	1:500	SC-22799	Santa Cruz Biotechnology	Anti-Rabbit (IgG)	1:1000	7074	Cell Signaling Techonology
BiP	1:1000	#3177	Cell Signaling Techonology	Anti-Rabbit (IgG)	1:2000	7074	Cell Signaling Techonology

Table S2. Antibody detail table.

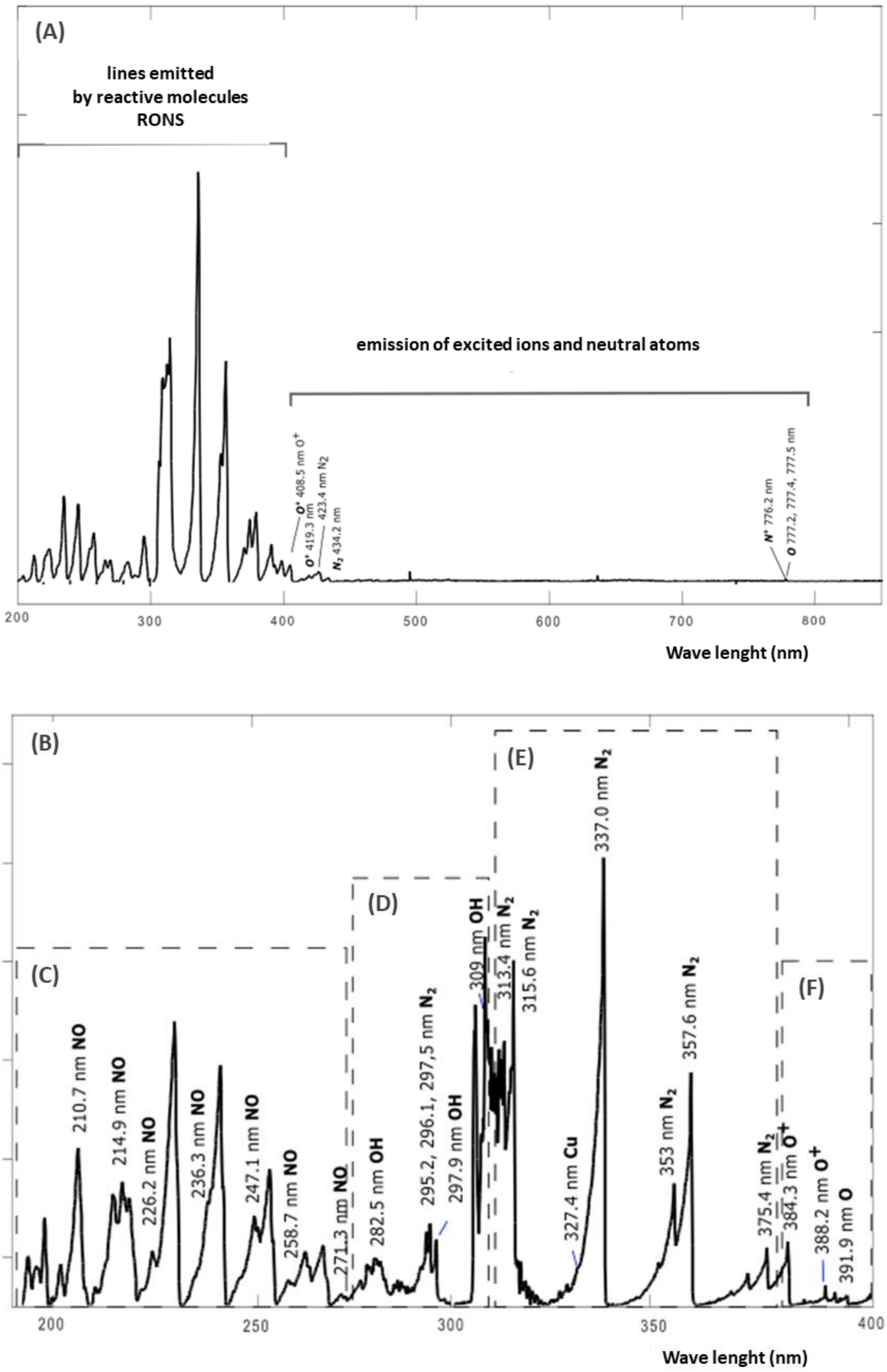

Figure S1. Optical emission spectroscopy (OES) spectrum. A. Complete spectrum observed in the wavelength range 200 to 800 nm corresponding to UV-VIS. B-F. Spectrum emission between 200 to 400nm image showing only in the ultraviolet range (UV) (B), where in the spectral lines of NO (nitric oxide) in the range from 200 to 275 nm (C), OH- (hydroxide ion) in the range from 275 to 310 nm (D), N2 (molecular nitrogen) in the range from 310 to 380 nm (E) and neutral and ionized atomic oxygen in the range from 380 to 400 nm (F).

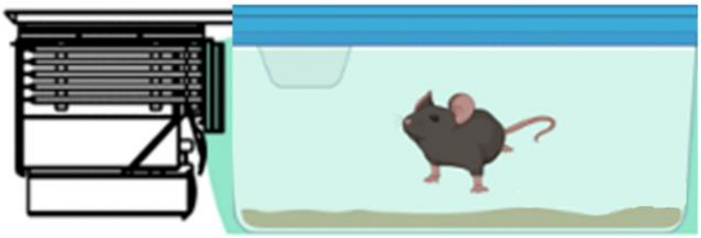

Figure S2. Scheme of the experimental arrangement.
